# Synergic action of an inserted carbohydrate-binding module in a glycoside hydrolase family 5 endoglucanase

**DOI:** 10.1107/S2059798322002601

**Published:** 2022-04-20

**Authors:** Ting-Juan Ye, Kai-Fa Huang, Tzu-Ping Ko, Shih-Hsiung Wu

**Affiliations:** a Institute of Biological Chemistry, Academia Sinica, Taipei 115, Taiwan; b Department of Chemistry, National Taiwan University, Taipei 115, Taiwan; c Institute of Biochemical Sciences, National Taiwan University, Taipei 115, Taiwan

**Keywords:** cellulases, cross-domain substrate binding, CBM insertion, *Mt*Glu5, *Meiothermus taiwanensis* WR-220

## Abstract

Most known cellulase-associated carbohydrate-binding modules (CBMs) are attached to the N- or C-terminus of the enzyme or are expressed separately and assembled into multi-enzyme complexes (for example to form cellulosomes), rather than being an insertion into the catalytic domain. Here, by solving the crystal structure, it is shown that *Mt*Glu5 from *Meiothermus taiwanensis* WR-220, a GH5-family endo-β-1,4-glucanase (EC 3.2.1.4), has a bipartite architecture consisting of a Cel5A-like catalytic domain with a (β/α)_8_ TIM-barrel fold and an inserted CBM29-like noncatalytic domain with a β-jelly-roll fold. Deletion of the CBM significantly reduced the catalytic efficiency of *Mt*Glu5, as determined by isothermal titration calorimetry using inactive mutants of full-length and CBM-deleted *Mt*Glu5 proteins. Conversely, insertion of the CBM from *Mt*Glu5 into *Tm*Cel5A from *Thermotoga maritima* greatly enhanced the substrate affinity of *Tm*Cel5A. Bound sugars observed between two tryptophan side chains in the catalytic domains of active full-length and CBM-deleted *Mt*Glu5 suggest an important stacking force. The synergistic action of the catalytic domain and CBM of *Mt*Glu5 in binding to single-chain polysaccharides was visualized by substrate modeling, in which additional surface tryptophan residues were identified in a cross-domain groove. Subsequent site-specific mutagenesis results confirmed the pivotal role of several other tryptophan residues from both domains of *Mt*Glu5 in substrate binding. These findings reveal a way to incorporate a CBM into the catalytic domain of an existing enzyme to make a robust cellulase.

## Abstract 2

A unique endoglucanase with a carbohydrate-binding module inserted in the middle of the catalytic domain has been characterized structurally and functionally, providing insights into the mode of action responsible for its enhanced catalytic performance.

## 1. Introduction

Plant cell walls (PCWs) are composed of polysaccharides and serve as an important resource for green materials and energy. Polysaccharide decomposition and utilization play a crucial role in nature, promoting progression of the carbon cycle. In industry, a limited supply of petroleum has increased the need for renewable energy sources such as biofuel products from PCWs (Kao *et al.*, 2019 ▸; Himmel *et al.*, 2007 ▸; Ragauskas *et al.*, 2006 ▸; Coughlan, 1985 ▸). The most abundant polysaccharide in PCWs, cellulose, consists of β-1,4-linked glucose, which can be degraded into mono/disaccharides before fermentation to bioethanol (Rubin, 2008 ▸; Demain *et al.*, 2005 ▸). Deconstruction processes of PCWs are generally mediated by glycoside hydrolases (GHs), which have been classified into different families in the carbo­hydrate-active enzymes (CAZy) database (Drula *et al.*, 2022 ▸; Lombard *et al.*, 2014 ▸). GHs that catalyse the hydrolysis of carbohydrates in PCWs, including cellulose and hemicellulose, also have industrial importance due to their wide applications (Gilbert, 2010 ▸; Himmel *et al.*, 2007 ▸; Kuhad *et al.*, 2011 ▸). Cellulases, which cleave the β-glycosidic bonds in cellulose, are mainly categorized into exo-glucanases, endo-glucanases and β-glucosidases. Exo- and endo-glucanases work together in a synergistic process in which exo-glucanases turn out cellobiose units from the end of crystalline regions of cellulose, while endo-glucanases work on amorphous regions of cellulose and create new ends for the exo-enzymes (Jalak *et al.*, 2012 ▸; Sakon *et al.*, 1997 ▸; Teeri, 1997 ▸).

The catalytic domain (CD) of cellulases contains the active site for hydrolysis, and the mode of action of the CD is based on its three-dimensional structure. For example, exo-cellulases have tunnel-like CD structures that only bind to the termini of cellulose molecules, whereas endo-cellulases usually have groove or cleft CD structures that bind to amorphous regions of cellulose (Horn *et al.*, 2012 ▸; Sakon *et al.*, 1997 ▸; Davies & Henrissat, 1995 ▸; Breyer & Matthews, 2001 ▸). On the other hand, noncatalytic carbohydrate-binding modules (CBMs) are found in many CAZymes and can provide specific interactions to help CAZymes act towards insoluble cellulose substrates (for example PCWs) in nature (Novy *et al.*, 2019 ▸; Sidar *et al.*, 2020 ▸; Hashimoto, 2006 ▸; Gilbert *et al.*, 2013 ▸; Hervé *et al.*, 2010 ▸; Boraston *et al.*, 2004 ▸; Tomme *et al.*, 1988 ▸). CBMs are grouped into three types based on their binding modes. Type A CBMs recognize and disrupt the surface of crystalline cellulose, type B CBMs bind individual polysaccharide chains in amorphous cellulose and type C CBMs have a lectin-like property that binds mono-, di- or trisaccharides (Gilbert *et al.*, 2013 ▸; Boraston *et al.*, 2004 ▸). CBMs commonly attach directly to CAZymes at their N- or C-terminal ends (Sidar *et al.*, 2020 ▸; Ravachol *et al.*, 2014 ▸; Urbanowicz *et al.*, 2007 ▸) or are assembled into cellulosome complexes (Eibinger *et al.*, 2020 ▸; Bayer *et al.*, 1998 ▸; Schwarz, 2001 ▸), thereby enhancing the enzyme–substrate interactions (Gilbert *et al.*, 2013 ▸; Venditto *et al.*, 2016 ▸; Hervé *et al.*, 2010 ▸; Luís *et al.*, 2013 ▸).

*Meiothermus taiwanensis* WR-220, a thermophilic, heterotrophic, aerobic Gram-negative bacterium isolated from Wu-rai hot springs in north Taiwan, has an optimum growth temperature of approximately 55°C (Chen *et al.*, 2002 ▸). Its importance is underscored by its industrial and biomedical applications, and it therefore has great potential for further exploration (Wu *et al.*, 2017 ▸; Liao *et al.*, 2013 ▸; Su *et al.*, 2016 ▸; Lin *et al.*, 2016 ▸). Several glycoside hydrolases were found in *M. taiwanensis* WR-220 by whole-genome sequencing, among which is a newly identified endo-β-1,4-glucanase. This enzyme, named *Mt*Glu5, belongs to subfamily 25 of glycoside hydrolase family 5 (GH5_25). GH5, which is also known as cellulase family A, is one of the largest GH families that hydrolyse major polysaccharide components in the biosphere (Aspeborg *et al.*, 2012 ▸; Henrissat *et al.*, 1989 ▸). *Mt*Glu5 contains a Cel5A-like CD similar to *Tm*Cel5A from *Thermotoga maritima* (Pereira *et al.*, 2010 ▸; Wu *et al.*, 2011 ▸), but has an additional novel domain inserted into an internal site in the CD. This novel domain, which was supposed to be a CBM and was thus named *Mt*CBM, shares low sequence identity with other known CBMs. Here, we determined crystal structures of full-length *Mt*Glu5 and CBM-deleted *Mt*Glu5 (dubbed ΔCBM) in apo and sugar-bound forms. We also characterized the biophysical properties of *Mt*Glu5 and investigated the effects of the presence or absence of the inserted CBM on the function of the CD. Furthermore, we inserted the CBM into *Tm*Cel5A to make a functional chimeric enzyme. Most importantly, this type of inserted CBM in an intact CD of a cellulase, although found in some xylanases (Flint *et al.*, 1997 ▸; Wu *et al.*, 2021 ▸), had never been investigated in detail before. Our structural and functional study of *Mt*Glu5 provides insight into a possible mode of action of the inserted CBM domain that could serve as a paradigm for thus far uncharacterized endo-cellulases with inserted CBMs from other microorganisms.

## 2. Methods

### 2.1. Cloning, expression and purification

The DNA fragment encoding residues 15–470 of *Mt*Glu5 (from which the signal peptide was removed) was amplified from the chromosomal DNA of *M. taiwanensis* WR-220 by PCR. The plasmids for ΔCBM (residues 1–235 and 366–455 of *Mt*Glu5), for site-directed mutagenesis of *Mt*Glu5 and for GST-fused g*Mt*CBM (residues 236–365 of *Mt*Glu5) were constructed using the *Mt*Glu5 gene as a template (Zeng, 1998 ▸). The gene fragment encoding *Tm*Cel5A was synthesized chemically, and the plasmid for *Tm*Cel5A-CBM expression was constructed using the *Tm*Cel5A and *Mt*Glu5 genes. The primers used are listed in Supplementary Table S1. All of the resultant DNA fragments were cloned into pET-21 vector with a C-terminal His_6_ tag. The recombinant plasmids were transformed into *Escherichia coli* BL21 (DE3) cells for protein expression. Inoculated competent cells were cultured in TB medium containing 50 µg ml^−1^ carbenicillin until the OD_600_ reached 1.0. Protein expression was then induced by adding isopropyl β-d-1-thiogalactopyranoside (IPTG; 1 m*M* final concentration) and the cells were further incubated at 37°C for 6 h. All recombinant proteins were purified by immobilized metal ion-affinity chromatography in lysis buffer (20 m*M* Tris, 100 m*M* sodium chloride, 20 m*M* imidazole pH 8). The elution used a 20–200 m*M* gradient of imidazole in the same buffer. Further purification by FPLC for crystallization and assays used Superdex 75 columns (GE Healthcare).

### 2.2. Crystallization and data collection

The inactive E393Q mutant, dubbed i*Mt*Glu5, was crystallized in the apo form by sitting-drop vapour diffusion by mixing equal volumes (1 µl) of protein solution (20 mg ml^−1^ in 20 m*M* Tris–HCl, 100 m*M* NaCl pH 8.0) and reservoir solution (0.1 *M* citric acid, 0.7 *M* ammonium sulfate, 20% PEG 400 pH 5.0) at room temperature. A sugar-bound co-crystal, *Mt*Glu5–glucose, was obtained in an MRC plate (Hampton Research, catalogue No. HR3-104) by the microbatch method (Chayen, 1997 ▸) using equal volumes (1 µl) of protein solution (30 mg ml^−1^ with a small amount of carboxymethyl cellulose, CMC) and reservoir solution (0.16 *M* citric acid, 1.1 *M* ammonium sulfate, 10% PEG 400 pH 5.0) under 10 µl Al’s oil (a 1:1 mixture of paraffin and silicone oil; Hampton Research, catalogue No. HR3-413). Apo-form CBM-deleted *Mt*Glu5, dubbed ΔCBM, was crystallized in an Eppendorf tube with a very high protein concentration (approximately 1500 µ*M* in 20 m*M* Tris, 100 m*M* NaCl pH 8.0) over a very long time (over a year) by evaporation. Sugar-bound crystals of ΔCBM were obtained by soaking ΔCBM crystals in a solution containing 10 m*M* cellobiose or cellopentaose. Glycerol was added as a cryoprotectant for all crystals prior to flash-cooling. The X-ray diffraction data sets were collected on beamlines 05A1, 15A1 and 13B1 at the National Synchrotron Radiation Research Center (NSRRC), Taiwan at 1.0 Å wavelength. All data sets were processed with *DENZO* in *HKL*-2000 (Otwinowski & Minor, 1997 ▸).

### 2.3. Structure determination and refinement

The crystal structure of i*Mt*Glu5 was solved by the molecular-replacement method using *MOLREP* (Vagin & Teplyakov, 2010 ▸) with the *Tm*Cel5A structure (PDB entry 3mmu; Pereira *et al.*, 2010 ▸) as a search model; the other structures were solved using the well refined i*Mt*Glu5 structure. The resulting model was subjected to computational refinement with *REFMAC*5 (Murshudov *et al.*, 2011 ▸). Well ordered solvent molecules, sugar ligands and water molecules were located with *Coot* (Emsley *et al.*, 2010 ▸). The stereochemical quality of the refined models was checked with *MolProbity* (Williams *et al.*, 2018 ▸). Final refinements were carried out by *Phenix* (Liebschner *et al.*, 2019 ▸) and some statistics are listed in Table 1 ▸. Molecular figures were produced with *PyMOL* (Schrödinger, USA). The coordinates and structure factors have been deposited in the Protein Data Bank with accession codes 7vt4, 7vt5, 7vt6, 7vt7 and 7vt8.

### 2.4. Isothermal titration calorimetry

The binding constant was determined using a Microcal iTC_200_ calorimeter (Malvern Panalytical) in Tris buffer (20 m*M* Tris, 100 m*M* NaCl pH 8.0). The titration experiments were performed by injecting 2 µl aliquots of 50 µ*M* CMC into a sample cell containing protein samples at 25–100 µ*M* at 25°C. The stirring speed and reference power were set to 750 rev min^−1^ and 5 µcal s^−1^, respectively. The reference heat background was determined under the same conditions by injecting CMC into buffer without protein. Thermodynamic parameters were calculated using Δ*G* = −*RT*ln*K*_a_ = Δ*H* − *T*Δ*S*, and data analysis was performed by the MicroCal *PEAQ-ITC* software version 1.21.

### 2.5. Enzyme activity assay

The reducing sugars produced from enzyme catalysis were determined using 3,5-dinitrosalicylic acid (DNS) reagent (Miller, 1959 ▸). The enzyme solution was mixed with carboxymethyl cellulose (CMC, 1%) and incubated for the given periods. The reactions were stopped by adding three times the volume of DNS reagent and boiling for 10 min. The absorbance at 540 nm was measured using a UV–Vis spectrophotometer (Infinite M1000 PRO, Tecan). The optimal conditions for *Mt*Glu5 and ΔCBM were determined at a range of pH values and temperatures in CHC buffer (20 m*M* citrate/HEPES/CHES, 100 m*M* NaCl). Enzyme activity assays on insoluble RAC substrates, which were produced from Avicel (Zhang, 2006 ▸), were performed under the optimal conditions for 30 min and then heated at 100°C for a further 10 min to stop the reaction, followed by adding three times the volume of DNS reagent and boiling for 10 min. After centrifugation to remove the pellet, the products were measured at an absorbance of 540 nm using a UV–Vis spectrophotometer. Glucose was used as a standard to produce the calibration curve. The activity was initially calculated as specific activity in IU, which is defined as micromoles of product per minute per micromole of protein, and presented as relative activity (the percentage of the activity of the wild-type *Mt*Glu5 enzyme) for easy comparison with the wild type.

The kinetic parameters of *Mt*Glu5 and its mutants towards the CMC substrate were obtained by fitting the initial reaction velocities at various substrate concentrations (which were varied from approximately 0.2 to 10 times the *K*_m_ of wild-type *Mt*Glu5) using the Michaelis–Menten equation in *GraphPad* 6.0. Each reaction was run at least in triplicate with seven substrate concentrations under the optimal conditions. The enzyme-kinetics assay was conducted according to previously described procedures (Liang *et al.*, 2018 ▸).

### 2.6. Substrate-binding model

The model was created according to superimposition of *Mt*Glu5 on *Tm*Cel5A in complex with cellotetraose and on CBM29-2 in complex with cellohexaose. Based on the cellotetraose and cellohexaose models, a polysaccharide chain composed of 14 glucose units was manually inserted into the *Mt*Glu5 structure. The model was then optimized by energy minimization.

### 2.7. Thermal shift assay

The binding of i*Mt*Glu5 and other mutants to CMC was verified by the intrinsic tryptophan and tyrosine fluorescence in a thermal shift assay using a Tycho NT.6 (NanoTemper Technologies) label-free differential scanning fluorimeter (Sierla *et al.*, 2018 ▸). The thermal stability of the inactive protein samples was measured in Tris buffer (20 m*M* Tris, 100 m*M* NaCl pH 8.0) with and without CMC. Aromatic residues buried in the protein core were exposed to the buffer upon temperature increase and protein unfolding. The shift of the 350/330 nm ratio was monitored in a quick thermal ramp from 35 to 95°C. The inflection point (*T*_i_) of protein unfolding was then determined.

## 3. Results and discussion

### 3.1. The crystal structure of *Mt*Glu5 shows an inserted CBM29-like domain

**Figure 1:**
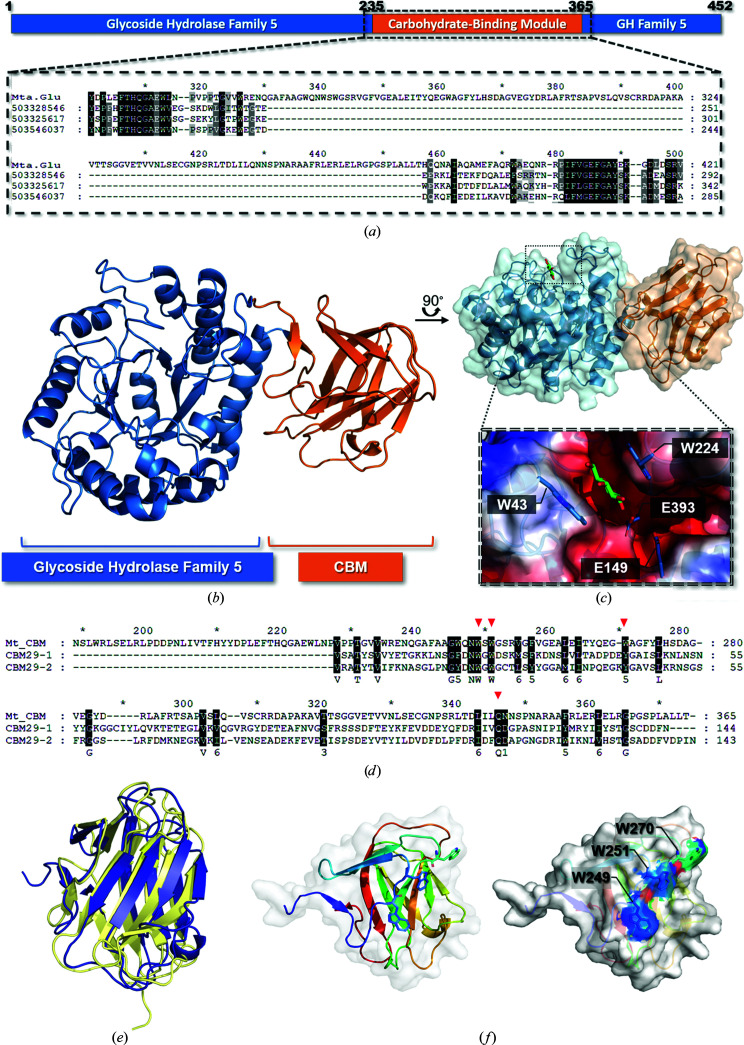
A novel endoglucanase from *M. taiwanensis* WR220 with an inserted carbohydrate-binding module (CBM). (*a*) Molecular architecture of *Mt*Glu5. The catalytic domain, which is shown in blue, belongs to glycoside hydrolase family 5, and the unique inserted CBM domain (orange) shares no homology with any other known GH5 sequence (Supplementary Fig. S1). (*b*) Structural overview of *Mt*Glu5 (PDB entry 7vt8). Cartoon depicting the 3D structure of the catalytic domain (blue) with a (β/α)_8_ TIM-barrel fold; the novel inserted CBM (orange) presents a β-jelly-roll fold. (*c*) The molecular electrostatic surface of *Mt*Glu5 in complex with a glucose molecule, which occupies the catalytic pocket. The glucose is sandwiched between the indole side chains of Trp43 and Trp224. The catalytic residues Glu149 and Glu393 are represented in stick format. (*d*) The amino-acid sequences of *Mt*CBM and CBM29. The *Mt*CBM sequence was aligned with CBM29 by *ClustalW*. The red triangles in the upper alignment indicate the conserved aromatic residues which are known to form the substrate-binding surface in CBM29; the red triangle in the lower alignment indicates the conserved glutamine residue for substrate recognition. (*e*) Overlap of *Mt*CBM (dark blue; PDB entry 7vt8) with CBM29-2 (pale yellow; PDB entry 1w8t), showing that the overall fold is similar in *Mt*CBM and CBM29-2. (*f*) The structure of *Mt*CBM colour-ramped from the N-terminus (blue) to the C-terminus (red), with the three conserved Trp residues which are on the solvent-exposed surface shown in stick format.

Analysis of the *Mt*Glu5 sequence by *BLASTp* on the NCBI website (https://www.ncbi.nlm.nih.gov/) indicated that *Mt*Glu5 belongs to the GH5 family but shares low overall identity with other cellulases in this family. However, using the multiple sequence alignment method of *ClustalW* (Thompson *et al.*, 1994 ▸), a unique inserted domain was identified in the middle of *Mt*Glu5 (Fig. 1 ▸ *a*). Further analysis of this domain alone with *BLASTp* revealed that it is probably a CBM. A model of the CD region was successfully predicted by *I-TASSER* (https://zhanggroup.org/I-TASSER/; Roy *et al.*, 2010 ▸) using *Tm*Cel5A (PDB entry 3mmu; Pereira *et al.*, 2010 ▸), which has approximately 50% identity to *Mt*Glu5, as a template, but the query coverage was only 64% and structure prediction failed for the new inserted domain. The model of the CD showed that Glu149 and Glu393 are the highly conserved general acid/base and nucleophile residues in the GH5 family. Initial attempts to crystallize wild-type *Mt*Glu5 failed. Instead, the inactive mutant E393Q (dubbed i*Mt*Glu5) was first crystallized in a suitable form for structure determination. The crystal structure was solved by the molecular-replacement approach using *Tm*Cel5A (PDB entry 3mmu) as a search model. The CDs of two i*Mt*Glu5 molecules were correctly located in the monoclinic *P*2_1_ unit cell. The initial map showed some density outside the CD regions, and amino-acid residues corresponding to the CBM were manually placed, followed by computational refinement and new map calculations. In this way, the model was gradually improved (Table 1 ▸).

Subsequently, sugar-bound crystals of wild-type *Mt*Glu5 were obtained by substrate co-crystallization with carboxy­methyl cellulose (CMC). On analysis of the cell content, the Matthews coefficient indicated that there might also be two *Mt*Glu5 molecules in the asymmetric unit of the tetragonal *I*422 crystal. However, it turned out to contain only one protein molecule in the asymmetric unit, with a high solvent content of ∼70%. In the refined model of *Mt*Glu5, the conserved CD has a (β/α)_8_ TIM-barrel fold and the inserted CBM shows a β-jelly-roll fold (Fig. 1 ▸ *b*, left). Bound glucose was observed in the *Mt*Glu5 crystal, whereas no sugar was seen in the i*Mt*Glu5 crystal (Fig. 1 ▸ *b*, right). Presumably, some CMC in the crystallization solution had been hydrolyzed by the active wild-type enzyme. Glucose was also found to be a product of CMC hydrolysis by *Tm*Cel5A (Basit & Akhtar, 2018 ▸). In the catalytic pocket, the glucose molecule is sandwiched between the indole side chains of Trp43 and Trp224, which are presumably engaged in stacking interactions. The binding site is adjacent to the two conserved catalytic residues Glu149 and Glu393 (Fig. 1 ▸ *c*). It corresponds to the −2 or −3 sugar of a bound cellulose substrate, according to the general nomenclature. The other sugar units were not observed because they were considered to be leaving products and were not properly retained in the active site (Wu *et al.*, 2011 ▸; Davies *et al.*, 1997 ▸).

To find out the possible classification of *Mt*CBM, *DALI* was used to search for similar structures (Holm & Sander, 1993 ▸; Holm, 2020 ▸). The search results showed that *Mt*CBM is topologically similar to CBM29-2, which belongs to the type B CBMs (Charnock *et al.*, 2002 ▸). Sequence alignment of *Mt*CBM and CBM29-2 revealed several conserved residues, including three surface aromatic amino acids Trp249, Trp251 and Trp270 in *Mt*CBM, which correspond to Trp24, Trp26 and Tyr46 in CBM29-2, respectively. Both the *Mt*CBM and the CBM29-2 structures show a β-jelly-roll topology, and they superimpose quite well with a root-mean-square deviation (r.m.s.d.) of 2.73 Å for 142 pairs of C^α^ atoms (Figs. 1 ▸ *d*–1*f*). The CBM29 family was first identified in *Piromyces equi* in 2001 (Freelove *et al.*, 2001 ▸). However, no other protein from a microorganism has shown homology to CBM29s in the CAZy database until now. According to the information on structural homology provided by the *DALI* server, the three surface tryptophan residues of *Mt*CBM are considered to play a key role in substrate binding, as are the corresponding aromatic residues in CBM29-2 (Charnock *et al.*, 2002 ▸; Flint *et al.*, 2004 ▸, 2005 ▸). Additionally, other non-aromatic sugar-binding residues in CBM29-2, such as Gln116, also have equivalents (Gln337) in *Mt*CBM, which are presumably engaged in similar inter­actions. The above structural analysis suggests a close kinship of *Mt*CBM to the CBM29 family. The precise placement of *Mt*CBM among the other known CBMs, however, awaits its classification by CAZy.

### 3.2. The presence of the *Mt*CBM domain greatly enhances the substrate affinity

**Figure 2:**
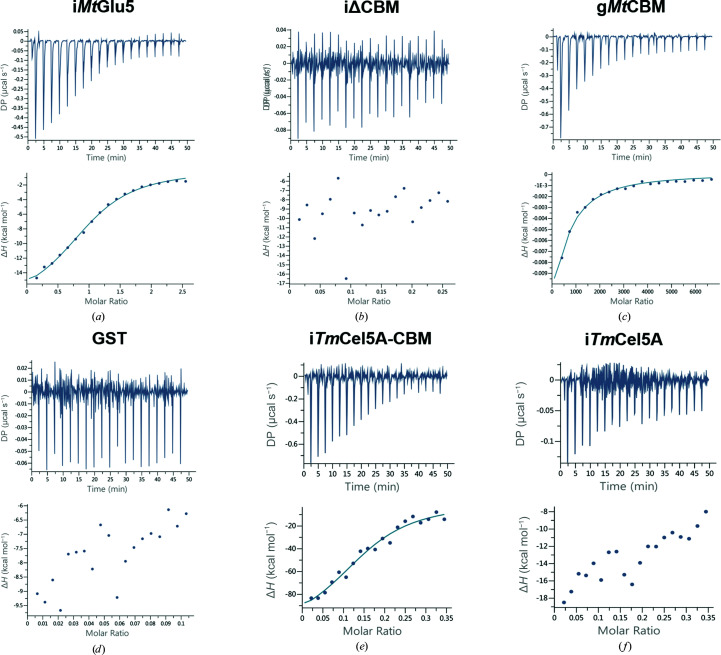
Representative ITC data for proteins binding to CMC. The top half of each panel shows the raw ITC heats of binding; the bottom half indicates the best fit of the integrated heats to a single-site model using the *PEAQ-ITC* software.

To characterize the function of the presumed *Mt*CBM (amino acids 236–365 of *Mt*Glu5), binding constants were determined by isothermal titration calorimetry (ITC). Inactive full-length *Mt*Glu5 (i*Mt*Glu5), inactive CBM-deleted *Mt*Glu5 (iΔCBM) and GST-fused *Mt*CBM (g*Mt*CBM) were constructed, expressed and purified for ITC experiments. The inactive proteins carried the active-site mutations E393Q in i*Mt*Glu5 and E263Q in iΔCBM to avoid interference in the ITC measurements. Despite numerous trials to purify *Mt*CBM alone under various conditions, the protein stability was too low for any further experiments. Therefore, a GST-fusion protein, dubbed g*Mt*CBM, was designed and the solubility problem was successfully solved. GST alone was also expressed and purified as a negative control. The binding profiles and calculated parameters for the four different proteins are shown in Figs. 2 ▸(*a*)–2 ▸(*d*) and Table 2 ▸. All data were fitted with a single-site binding model. Because the CMC titrant and its molar concentration of binding sites are unknown, the *n* value was modified to 1 together with adjusting the concentration of the titrant (Szabó *et al.*, 2001 ▸; Carvalho *et al.*, 2004 ▸; Campos *et al.*, 2016 ▸). The data show that i*Mt*Glu5 has a strong affinity for the recognition of long single polysaccharide chains with *K*_d_ = 5.71 × 10^−6^ (Fig. 2 ▸ *a*). However, the data for iΔCBM showed no significant binding to the CMC substrate, confirming that the deleted domain is responsible for substrate binding (Fig. 2 ▸ *b*). Moreover, the data for g*Mt*CBM also demonstrated significant affinity, with *K*_d_ = 4.56 × 10^−2^, while in contrast GST displayed no binding (Figs. 2 ▸ *c* and 2 ▸ *d*). The calculated thermodynamic parameters indicated that the binding between *Mt*CBM and the polysaccharide chain was enthalpically favorable, similar to most other protein–carbohydrate interactions, including those observed in type B CBMs.

Furthermore, to investigate the possible enhancement of the activity of other enzymes on incorporating *Mt*CBM, we constructed a chimeric *Tm*Cel5A-CBM protein in which *Mt*CBM was inserted into *Tm*Cel5A at an equivalent internal location to that in *Mt*Glu5. For comparative purposes, *Tm*Cel5A alone was also expressed and purified. Again, to avoid interference, subsequent ITC measurements used the inactive E253Q mutant of *Tm*Cel5A, and the corresponding proteins were named i*Tm*Cel5A and i*Tm*Cel5A-CBM. As expected, significantly different binding profiles were observed (Figs. 2 ▸ *e* and 2 ▸ *f*): i*Tm*Cel5A displayed low affinity towards CMC, similar to iΔCBM, while in contrast the chimeric i*Tm*Cel5A-CBM exhibited a strong affinity like that of i*Mt*Glu5 (Table 2 ▸). The results of the ITC experiment serve as evidence of synergy between the CD and CBM in *Mt*Glu5, as well as in the chimeric *Tm*Cel5A-CBM. Full-length i*Mt*Glu5 presents an approximately 10^5^-fold higher affinity towards substrate than the individual iΔCBM or g*Mt*CBM (Table 2 ▸ and Fig. 2 ▸). This synergistically cooperative binding between two domains has also been demonstrated in other CBM-associated proteins such as CBM29-1-2 and Starch Excess4 (SEX4), but is not always present (Freelove *et al.*, 2001 ▸; Meekins *et al.*, 2014 ▸; Fox *et al.*, 2013 ▸; Fernandes *et al.*, 1999 ▸; Várnai *et al.*, 2013 ▸). Presumably, the inserted CBMs act in synergy with CDs just like the intact i*Mt*Glu5 and the chimeric i*Tm*Cel5A-CBM, which display an impressively higher affinity for the soluble substrates.

### 3.3. Deletion of the CBM domain severely impairs the catalytic efficiency of *Mt*Glu5

**Figure 3:**
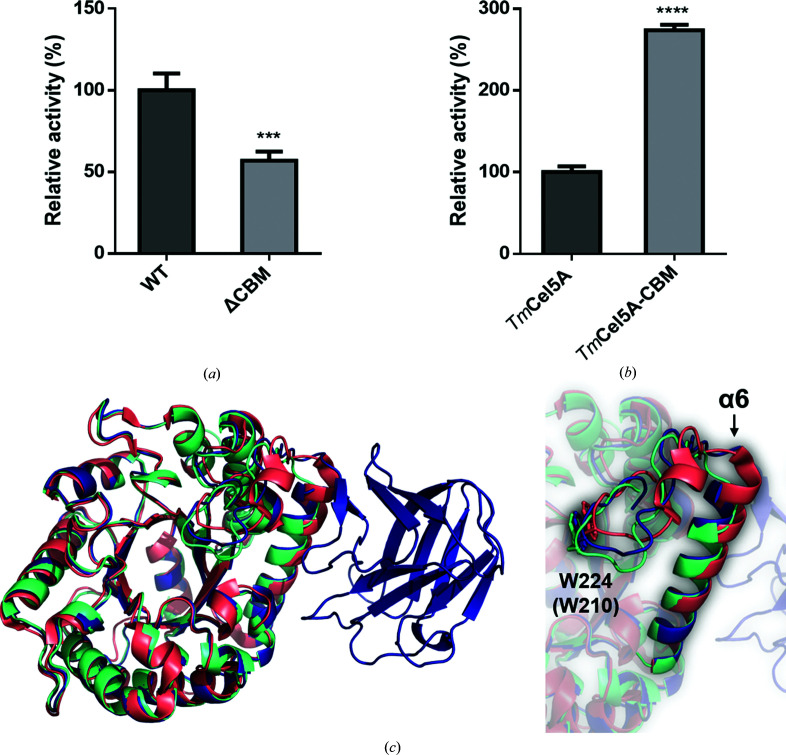
Functional and structural comparison among enzymes with or without an inserted *Mt*CBM. (*a*) Comparison of the glucanase activity of *Mt*Glu5 (WT) and ΔCBM towards regenerated amorphous cellulose (RAC). The specific activities of *Mt*Glu5 and ΔCBM towards RAC are 4.24 ± 0.44 and 2.41 ± 0.24 IU, respectively. The activities are presented as relative activity (%) measured in optimum buffer consisting of 20 m*M* citrate/HEPES/CHES and 100 m*M* sodium chloride pH 5.0. Data are exhibited as the means ± SD of more than three replicates. *** indicates statistical significance at the *p* < 0.001 level compared with *Mt*Glu5. (*b*) Comparison of the glucanase activity of wild-type *Tm*Cel5A and chimeric *Tm*Cel5A-CBM towards RAC under optimal conditions. The specific activities of *Tm*Cel5A and *Tm*Cel5A-CBM towards RAC are 1.57 ± 0.11 and 4.30 ± 0.10 IU, respectively. The activities are presented as the relative activity (%) measured under optimum conditions. Data are exhibited as the means ± SD of more than three replicates. **** indicates statistical significance at the *p* < 0.0001 level compared with *Tm*Cel5A. (*c*) Superimposition of the structures of *Mt*Glu5 (dark blue; PDB entry 7vt8), ΔCBM (green cyan; PDB entry 7vt5) and *Tm*Cel5A (deep salmon; PDB entry 3mmu). Each catalytic domain of the three structures overlays well except for the α6 helix and the loop containing an important residue for substrate binding: Trp224 in *Mt*Glu5 and ΔCBM (equivalent to Trp210 in *Tm*Cel5A).

The overall structure of *Mt*Glu5 suggests clear separation of the CD and CBM into two independent domains (Fig. 1 ▸ *b*). To better determine the role of the noncatalytic *Mt*CBM, the active-form proteins full-length *Mt*Glu5 and CBM-deleted ΔCBM were assayed for enzymatic activity. After testing various conditions, subsequent measurements were performed in 20 m*M* CHC buffer pH 5.0 with incubation for 10 min at 60°C (Supplementary Fig. S2). The results using the insoluble substrate regenerated amorphous cellulose (RAC) are shown in Fig. 3 ▸(*a*). The enzymatic activity of ΔCBM towards the RAC substrate was reduced to 55% of that of *Mt*Glu5 (*P* < 0.001). Similar results were obtained using the soluble substrate CMC (Supplementary Fig. S3). The kinetic data indicated that without the CBM domain, the *K*_m_ of ΔCBM increased nearly ninefold and the *k*_cat_ decreased by more than tenfold (Table 3 ▸; Supplementary Fig. S4*a* ). As a comparison, we also measured the enzymatic activity of *Tm*Cel5A and *Tm*Cel5A-CBM against RAC and determined the *K*_m_ and *k*_cat_ towards CMC. As shown in Fig. 3 ▸(*b*), incorporation of the CBM tripled the overall activity of *Tm*Cel5A towards RAC (*p* < 0.0001), which was mainly a result of the decreased *K*_m_, as the *k*_cat_ largely remained the same (Table 3 ▸; Supplementary Fig. S4*b* ). Interestingly, the *K*_m_ values of *Tm*Cel5A and ΔCBM are very similar, suggesting a shared low substrate affinity of the catalytic domain. Nevertheless, the artificial *Tm*Cel5A-CBM had a much lower performance when compared with the natural *Mt*Glu5, presumably due to a lack of optimization. In fact, the first attempt to insert *Mt*CBM into *Tm*Cel5A at a site just ten residues away failed to improve its activity, providing evidence for the importance of precise binding-cleft alignment with the CD for an inserted CBM to be beneficial.

In most other studies, CBM deletion or translocation did not dramatically change the activities of endo-cellulases (Wu *et al.*, 2018 ▸; Várnai *et al.*, 2013 ▸). Likewise, *Tm*Cel5A had almost the same *k*_cat_ as *Tm*Cel5A-CBM. However, ΔCBM showed a dramatically lower enzymatic activity than *Mt*Glu5 (Fig. 3 ▸ *a*, Supplementary Fig. S3). The lower enzymatic activity of ΔCBM is not due to structural damage to the catalytic center, the integrity of which was demonstrated by analyzing the hydrolyzed products using mass spectroscopy. Both full-length and CBM-deleted *Mt*Glu5 remain capable of hydrolysis (Supplementary Fig. S5). To investigate the origin of the disparity in catalytic efficiency between ΔCBM and *Mt*Glu5, we crystallized ΔCBM and solved the structure by molecular replacement using the CD of *Mt*Glu5 as a search model. The orthorhombic *P*2_1_2_1_2_1_ crystal contained one molecule of ΔCBM in its asymmetric unit. The apo-form structure of ΔCBM displayed an intact TIM-barrel fold (Supplementary Fig. S6). The crystal structure of ΔCBM presents almost the same polypeptide conformation as observed in the CD of *Mt*Glu5, with an r.m.s.d. of 0.28 Å for 260 pairs of C^α^ atoms (Fig. 3 ▸ *c*, left). Both structures superimpose well with *Tm*Cel5A, with r.m.s.d.s of 0.56 and 0.54 Å for 263 and 261 C^α^ atom pairs, respectively (Fig. 3 ▸ *c*, left). Moreover, ΔCBM crystals in complex with glucose and cellobiose were obtained by soaking with cellopentaose and cellobiose, respectively. The sugar molecules in both structures were also sandwiched between the Trp43 and Trp224 side chains just as in *Mt*Glu5 (Supplementary Figs. S7 and S8). The presence of bound glucose rather than cellopentaose in the former crystal is also evidence that ΔCBM remains capable of catalysis. The much lower *k*_cat_ value of ΔCBM was likely to be a result of loop perturbation. In *Mt*Glu5 (and also in *Tm*Cel5A) the β6–α6 loop is 30 residues long and contains the substrate-binding Trp224 (Trp210). Direct elimination of the CBM from *Mt*Glu5 resulted in a shorter connection between this loop and the α6 helix, the equivalent of which in *Tm*Cel5A has one additional turn (Fig. 3 ▸ *c*, right). Although the overall fold remained intact, the lack of flexibility in the tightened-up loop would probably have reduced the catalytic efficiency of ΔCBM.

### 3.4. The complex model reveals a substrate-binding cleft lined with aromatic residues

**Figure 4:**
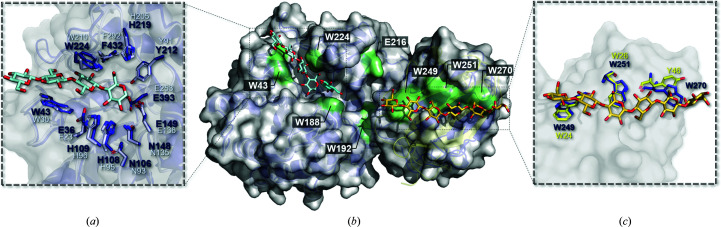
The structure of *Mt*Glu5 in comparison with those of *Tm*Cel5A and CBM29-2. (*a*) Superimposition of the active-site residues in the catalytic pocket of *Mt*Glu5 (dark blue) on the corresponding site of *Tm*Cel5A in complex with cellotetraose and glucose (blue/white; PDB entries 3azt and 3azr), depicted in stick representation, reveals the conservation of amino-acid residues between *Mt*Glu5 and *Tm*Cel5A. Likewise, in an overlay of the structure of *Mt*CBM (dark blue) and CBM29-2 in complex with cellohexaose (yellow; PDB entry 1w8t), with the three conserved aromatic residues shown in stick format, reveals substrate binding by *Mt*CBM in a similar way to CBM29-2 (*c*). (*b*) A putative binding groove of *Mt*Glu5 with many Trp residues on the solvent-exposed hydrophobic surface: Trp43, Trp188, Trp192, Trp224, Trp249, Trp251 and Trp270 are shown together with Glu216 for comparison.

According to the above structural data, a plausible substrate-binding model of *Mt*Glu5 can be constructed. Inspection of the *Mt*Glu5 structure shows a distinct cleft that traverses from the bound sugars on one side of the CD through the catalytic pocket to the CBM domain on the other side. It is presumed to be a possible substrate-binding groove with many surface tryptophan residues. *Mt*Glu5 consists of two domains: a Cel5A-like TIM-barrel fold catalytic domain and a CBM29-2-like noncatalytic carbohydrate-binding module which is inserted into the intact CD and extends the binding groove (Fig. 4 ▸). To investigate the possible substrate-recognition mechanism, the structure of *Mt*Glu5 was compared with those of *Tm*Cel5A and CBM29-2. Superimposition of *Tm*Cel5A in complex with cellotetraose onto the CD of *Mt*Glu5 showed that the residues in the catalytic pocket overlaid very well, revealing highly conserved structures (Fig. 4 ▸ *a*). Both of them share a similar substrate-binding cleft beside the catalytic pocket (Fig. 4 ▸ *b*). Likewise, when CBM29-2 in complex with cellohexaose was superimposed on *Mt*CBM, the three conserved aromatic residues on the hydrophobic surface of CBM29-2 overlaid well with *Mt*CBM, except for Tyr46 and Trp270. The superimposition also revealed an ambiguous binding cleft in *Mt*CBM through the three corresponding surface aromatic residues (Fig. 4 ▸ *c*). Combined with the superimposition results of *Mt*Glu5, *Tm*Cel5A in complex with cellotetraose and CBM29-2 in complex with cellohexaose, a plausible continuous binding groove emerged that is richly embedded with surface tryptophan residues (Fig. 4 ▸ *b*). The affinity and kinetic data also indicated that both the CD and CBM of *Mt*Glu5 contribute to its binding to polysaccharide substrates.

Although only a couple of bound sugar residues were seen in the CD of *Mt*Glu5, their positions are consistent with those in *Tm*Cel5A. While no sugar was seen in the CBM, the conserved surface aromatic residues suggest a similar sugar-binding mode to those of CBM29s. A model was therefore generated by bridging the segments of bound sugars from *Tm*Cel5A and CBM29-2 with three additional residues, making a continuous polysaccharide chain that passes through the putative binding groove (Supplementary Fig. S9*a* ). The model was subjected to energy minimization using the *YASARA* server (http://www.yasara.org/minimizationserver.htm; Krieger *et al.*, 2002 ▸). In the CD of this model, the three important tryptophan residues Trp43, Trp224 and Trp188, as well as other active-site residues, for example His108, His109 and Tyr212, can form hydrogen bonds to the substrate in the catalytic pocket (Supplementary Fig. S9*b* ). Additionally, *Mt*CBM may make hydrophobic and van der Waals inter­actions with the substrate through the three crucial surface tryptophan residues Trp249, Trp251 and Trp270. The backbone of Trp249 and the side chains of Arg303 and Gln337 may also form direct hydrogen bonds to the substrate (Supplementary Fig. S9*c* ). In this model, the putative binding groove is 12–14 saccharides in length from one side to the other. Moreover, numerous factors may contribute to carbohydrate binding. Some of the pivotal factors, for example stacking interactions, are provided by aromatic residues. The stacking interaction of aromatic residues against the pyranose ring of sugars consists of hydrophobic interactions, van der Waals interactions, hydrogen bonding and even CH–π interaction of aromatic residues in CBMs (Campos *et al.*, 2016 ▸; Szabó *et al.*, 2001 ▸; Carvalho *et al.*, 2004 ▸; Venditto *et al.*, 2016 ▸; Nagy *et al.*, 1998 ▸; Flint *et al.*, 2004 ▸; Asensio *et al.*, 2013 ▸). Tryptophan is usually the most essential residue to provide stacking interactions with sugar residues in other known CBMs. However, in the complex structure of *Mt*Glu5 and the proposed binding model tryptophan residues are not only present in the substrate-binding groove of CBM but also in the CD region. In other words, these surface aromatic residues are presumed to work together in binding to the polysaccharide substrate.

Similar cross-domain binding to the substrate has been found in, for example, the processive endoglucanase E4 (now Cel9A-68) from *Thermomonospora fusca*, which also shows an extended binding surface from E4_CD_ to the C-terminal CBM3c (Sakon *et al.*, 1997 ▸). Previous studies demonstrated that the type A CBM3c is essential for crystalline cellulose binding and catalytic processivity in Cel9A-68. However, the flat binding surface of CBM3c lacks several conserved aromatic residues which are important for substrate binding in CBM3a and CBM3b. Furthermore, mutagenesis of the binding-surface residues on CBM3c does not dramatically decrease the activity of Cel9A-68 (Li *et al.*, 2007 ▸, 2010 ▸; Tormo *et al.*, 1996 ▸). Unlike the processive endoglucanase Cel9A-68, neither *Mt*Glu5 nor ΔCBM present processivity, indicating that the extended binding groove in the presence of a type B *Mt*CBM does not contribute to processive hydrolysis (Supplementary Table S2; Irwin *et al.*, 1993 ▸). Moreover, *Mt*Glu5 presents synergistic cooperation between the CD and CBM in which each tryptophan side chain on the binding groove is essential for hydrolytic activity (see below). This is also different from Cel9A-68. Although the substrate-binding modes appear to be similar for *Mt*Glu5 and Cel9A-68, the biochemical properties of the two enzymes are distinct, indicating that *Mt*Glu5 has a novel binding mode compared with Cel9A-68.

### 3.5. Mutagenesis results support the key role of aromatic residues in substrate binding

**Figure 5:**
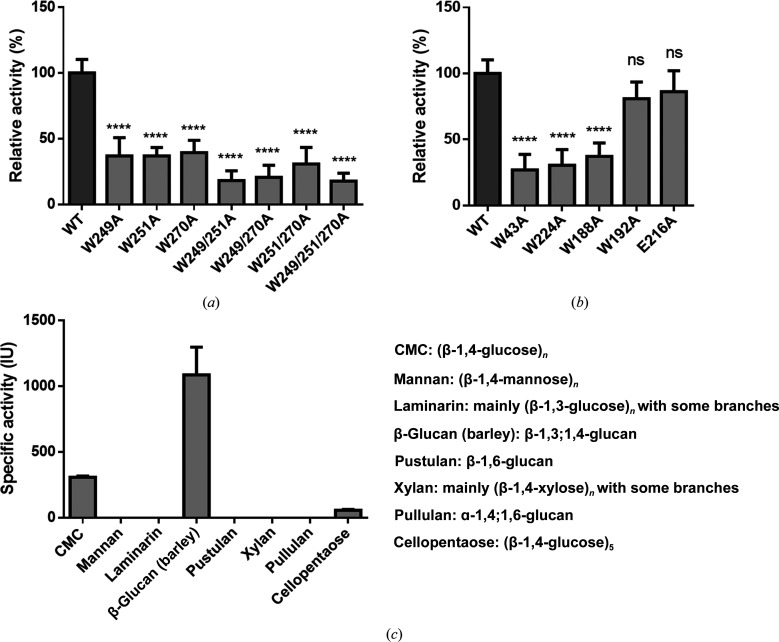
Comparison of the activity of *Mt*Glu5 and Trp mutants towards RAC. (*a*) Single to triple Trp mutations of *Mt*CBM. In contrast to the WT, all Trp mutants of *Mt*CBM show decreased glucanase activity. (*b*) The single amino-acid mutations in the putative binding groove of the catalytic domain, W43A, W188A and W224A, show dramatically decreased activity. However, the W192A and E216A mutants in the interactive surface between the CD and CBM display no significant difference. All activities are presented as relative activity (%) measured at optimum pH and temperature. Data are shown as the means ± SD of more than three replicates. **** indicates statistical significance at the *p* < 0.0001 level and ns represents no statistical significance compared with *Mt*Glu5. (*c*) Enzyme activities of *Mt*Glu5 towards various substrates. As the data show, *Mt*Glu5 is highly specific for β-1,4-glucan, indicating that *Mt*Glu5 belongs to EC 3.2.1.4. The specific activities of *Mt*Glu5 towards CMC, β-glucan (barley) and cellopentaose are 385 ± 5, 1090 ± 210 and 56.5 ± 6.5 IU, respectively.

By structural comparison with CBM29-2, the three surface tryptophan residues Trp249, Trp251 and Trp270 in *Mt*CBM were presumed to participate in substrate binding. Here, this hypothesis was verified by site-directed mutagenesis. The glucanase activity towards RAC of the single Trp mutants W249A, W251A and W270A, the double Trp mutants W249/251A, W249/270A and W251/270A and the triple Trp mutant W249/251/270A in the CBM region of *Mt*Glu5 were measured and compared with the wild-type enzyme (Fig. 5 ▸ *a*). All of the single mutants retained ∼40% of the activity of *Mt*Glu5. Moreover, the data for the double mutants and triple mutant showed that when Trp249 and Trp251 were mutated simultaneously, the activity was dramatically reduced by ∼80%. In either the single, double or triple mutants, altering Trp270 seems to have milder adverse effects, probably due to its distal location in the substrate-binding cleft. Similar results were obtained using CMC as the substrate (Supplementary Fig. S10*a* ). Four other surface tryptophan residues, Trp43, Trp224, Trp188 and Trp192, are found in the putative binding groove of the CD of *Mt*Glu5. These four residues, along with the nearby non-aromatic Glu216, which served as a negative control, were also tested by site-directed mutagenesis. Using RAC as the substrate, W43A, W224A and W188A showed a prominent reduction in activity to less than 50%, whereas the effects of W192A and E216A were not as significant (Fig. 5 ▸ *b*, Supplementary Fig. S10*b* ).

Further kinetic measurements showed that single Trp mutants in the CBM drastically increased the *K*_m_ to 15–17 times that of the wild type (Table 3 ▸, Supplementary Fig. S4*c* ), but the *k*_cat_ was also increased by 2.2–3.6-fold. The reduced substrate affinity might facilitate product release, resulting in higher turnover numbers. However, the double and triple Trp mutants did not show a further increase in *K*_m_, whereas the *k*_cat_ values were significantly reduced. The resulting *k*_cat_/*K*_m_ values were comparable to that of ΔCBM, suggesting that these mutants virtually aborted the original function of the CBM. Again, mutants involving the distal Trp270 showed milder effects on the activity of the enzyme (Fig. 5 ▸ *a*, Supplementary Fig. S10*a* ). The *K*_m_ values of the W43A, W188A, W192A and E216A mutants in the CD region did not increase as much as those of the CBM mutants described above, but the *K*_m_ of W224A was eight times that of the wild type (Table 3 ▸, Supplementary Fig. S4*d* ). On the other hand, only the *k*_cat_ of W43A and W224A showed a prominent reduction, by threefold and fourfold, respectively. Both Trp43 and Trp224 participate in binding to sugar units on the nonreducing end of the polysaccharide substrate immediately adjacent to the active site, and their absence should slow the reaction (Fig. 1 ▸ *c*, Table 3 ▸). The low *k*_cat_/*K*_m_ value of W224A again underscores the key role of Trp224 in catalysis by *Mt*Glu5. In comparison, the sustained activity of W188A shows that Trp188 is less important in substrate binding. In comparison, the activity of W192A and E216A was only slightly decreased. Trp192 is indeed located at the CD–CBM interface, and Glu216 is in the Trp224-containing β6–α6 loop. Both residues apparently do not interact directly with the substrate.

Interestingly, by testing with various other substrates, *Mt*Glu5 was found to be more active towards β-glucan than CMC, indicating that the enzyme may also bind efficiently to mixed 1,3- and 1,4-linked glucose units (Fig. 5 ▸ *c*). However, the enzyme showed only β-1,4-glucanase activity. It did not cleave branched or α-linked glucans and nonglucose substrates. Additionally, ligand-induced protein stabilization is a widely known phenomenon that has been well described (Pace & McGrath, 1980 ▸; Pantoliano *et al.*, 2001 ▸). To further evaluate the importance of tryptophan residues in substrate binding, the thermal shift assay, or the inflection point of protein unfolding (Δ*T*_i_), was measured for each inactive mutant in the presence or absence of CMC. As shown in Table 4 ▸, stabilization of i*Mt*Glu5 by CMC binding resulted in a Δ*T*_i_ of 4°C. The diminished thermal shift of iΔCBM indicates that *Mt*CBM plays a key role in substrate binding. Similar effects were seen for Trp249 and Trp251 mutations in the CBM region. Mutation of the distal Trp270 in CBM had a less prominent effect, and so did those of the other tryptophan residues Trp43, Trp188 and Trp224 in the CD region, suggesting minor but still essential roles in substrate binding (Table 4 ▸). The thermal shift data are consistent with the above catalytic activity measurements, while mutating Trp192 and Glu216 showed milder effects on the activity but even larger Δ*T*_i_ values than the wild-type enzyme (Table 4 ▸), suggesting a close relationship between substrate binding and a stable functioning protein conformation.

## 4. Concluding remarks

In this study, we report a new type of endoglucanase with a novel inserted CBM that is in the middle of the intact CD. Unlike other known cellulases with CBMs at the termini, this inserted CBM in the newly identified *Mt*Glu5 acts in synergy with the CD, as demonstrated by ITC experiments. Superimposition of *Mt*Glu5 with homologous structures, namely *Tm*Cel5A and CBM29-2, and the subsequent construction of a substrate-binding model suggest that the polysaccharide chain interacts with a continuous groove extending from the CD to the CBM. The binding mode of the endoglucanase *Mt*Glu5 is similar but not the same as that of Cel9A-68 from *T. fusca*. Six tryptophan residues in the groove play a key role in sugar binding: Trp249, Trp251 and Trp270 in the CBM, and Trp43, Trp224 and Trp188 in the CD. As demonstrated by mutagenesis experiments, the loss of these surface tryptophans significantly reduced the substrate affinity. We also showed that by extending the substrate-binding groove, the chimeric *Tm*Cel5A-CBM was endowed with a higher affinity for longer polysaccharides. Notably, the inserted CBM is not a fixed attachment, as its orientation varies with respect to the CD in the monoclinic and tetragonal crystals of i*Mt*Glu5 and *Mt*Glu5. The CBMs of the two i*Mt*Glu5 molecules differ by a rigid-body rotation of 22.5°, and they differ from that of wild-type *Mt*Glu5 by rotations of 25.3° and 10.8° (Supplementary Fig. S11). While large-scale conformational changes are often observed in enzymes upon substrate binding (Arora & Brooks, 2007 ▸; Suzuki *et al.*, 2019 ▸), the moderate flexibility of CBM in *Mt*Glu5 probably allows switching between different orientations of polysaccharide chains. The above findings provide a new paradigm for studying other as yet uncharacterized cellulases with inserted CBMs, which might act against natural insoluble substrates more efficiently in potential industrial applications. This approach may be complementary to recent work on multi-enzyme complexes in cellulose deconstruction (McConnell *et al.*, 2020 ▸).

## Supplementary Material

PDB reference: *Mt*Glu5, E393Q mutant, 7vt4

PDB reference: complex with glucose, 7vt8

PDB reference: ΔCBM, 7vt5

PDB reference: complex with glucose, 7vt6

PDB reference: complex with cellobiose, 7vt7

Supplementary Figures and Tables. DOI: 10.1107/S2059798322002601/jc5049sup1.pdf

## Figures and Tables

**Table 1:** Data-collection and refinement statistics

	*Mt*Glu–glucose	i*Mt*Glu	ΔCBM	ΔCBM–glucose	ΔCBM–cellobiose
Data collection					
Wavelength (Å)	1.0	1.0	1.0	1.0	1.0
Resolution (Å)	50–2.99	50–1.90	50–1.46	50–1.53	50–1.53
Space group	*I*422	*P*2_1_	*P*2_1_2_1_2_1_	*P*2_1_2_1_2_1_	*P*2_1_2_1_2_1_
*a*, *b*, *c* (Å)	144.93, 144.93, 197.57	74.25, 114.35, 80.15	49.55, 75.35, 82.15	49.68, 75.23, 83.47	49.73, 75.29, 83.39
α, β, γ (°)	90.0, 90.0, 90.0	90.00, 103.23, 90.00	90.00, 90.00, 90.00	90.00, 90.00, 90.00	90.00, 90.00, 90.00
Total observations	168758	373384	189709	190955	173135
Unique reflections	21603 (1055)	97424 (9740)	51808 (4780)	45521 (4725)	46830 (4737)
Multiplicity	7.8 (6.3)	3.8 (3.8)	3.7 (3.0)	4.2 (4.6)	3.7 (3.9)
Completeness (%)	99.8 (99.1)	99.9 (100.0)	96.0 (90.3)	94.8 (100.0)	97.3 (99.9)
〈*I*/σ(*I*)〉	26.55 (1.29)	22.06 (2.00)	26.14 (2.56)	33.82 (6.56)	31.66 (4.29)
*R*_merge_ (%)	7.1 (104.2)	5.9 (74.9)	5.7 (50.3)	5.3 (18.6)	5.2 (26.0)
Refinement					
Resolution (Å)	29.215–2.987 (3.093–2.987)	26.974–1.930 (1.999–1.930)	24.221–1.459 (1.512–1.459)	24.276–1.529 (1.584–1.529)	27.942–1.529 (1.584–1.529)
No. of reflections	21515 (2058)	87371 (4143)	51733 (4548)	45450 (4693)	46752 (4706)
*R*_work_/*R*_free_	0.2055/0.2490	0.1581/0.1968	0.1480/0.1743	0.1485/0.1751	0.1480/0.1746
R.m.s.d., bond lengths (Å)	0.002	0.008	0.011	0.0085	0.0096
R.m.s.d., angles (°)	0.52	0.95	1.15	1.11	1.10
No. of atoms					
Protein	3518	7033	2625	2575	2560
Sugar	12	—	—	12	23
Glycerol	—	36	—	12	12
Water	36	1550	495	539	558
Average *B* factor (Å^2^)					
Protein	118.1	22.4	18.0	15.9	15.9
Sugar	145.9	—	—	37.6	35.2
Glycerol	—	46.6	—	29.9	29.4
Water	90.1	43.1	37.7	29.8	30.3
Ramachandran plot (%)					
Favored	94.48	97.01	97.09	97.41	97.09
Allowed	5.06	2.76	2.59	2.27	2.59
Outliers	0.46	0.23	0.32	0.32	0.32
PDB code	7vt8	7vt4	7vt5	7vt6	7vt7

**Table 2:** Affinity of proteins for polysaccharides determined by ITC

Protein	*K*_d_	Δ*G* (kcal mol^−1^)	Δ*H* (kcal mol^−1^)	*T*Δ*S* (kcal mol^−1^)	*n* †
i*Mt*Glu5	(5.71 ± 0.44) × 10^−6^	−7.15	−18.3	−11.2	1.00
iΔCBM	NB‡	—	—	—	—
g*Mt*CBM	(4.50 ± 0.27) × 10^−2^	−1.83	−16.2	−14.4	0.94
GST	NB	—	—	—	—
i*Tm*Cel5A§	NB	—	—	—	—
i*Tm*Cel5A-CBM	(8.20 ± 2.05) × 10^−6^	−6.94	−17.5	−10.5	1.00

† Number of binding sites on the protein.

‡ No binding detected.

§ Binding too weak to quantify by ITC.

**Table 3:** Kinetic parameters of *Mt*Glu5 and *Tm*Cel5A series towards CMC Kinetic parameter estimates ± SE (*n* = 3).

	*K*_m_ (mg ml^−1^)	*k*_cat_ [µmol (µmol protein)^−1^ s^−1^]	*k*_cat_/*K*_m_ [µmol (µmol protein)^−1^ s^−1^/(mg ml^−1^)]
WT	3.91 ± 1.36	268.58 ± 28.70	68.71 ± 21.09
ΔCBM	34.25 ± 11.11	26.33 ± 5.06	0.77 ± 0.46
*Tm*Cel5A	36.22 ± 12.96	107.67 ± 23.22	2.97 ± 1.79
*Tm*Cel5A-CBM	18.93 ± 6.27	100.13 ± 16.25	5.29 ± 2.59
Single Trp mutations of CBM			
W249A	58.00 ± 10.03	594.35 ± 70.80	10.25 ± 7.06
W251A	67.20 ± 15.83	678.98 ± 114.42	10.10 ± 7.23
W270A	62.97 ± 17.52	953.90 ± 185.18	15.15 ± 10.57
Double Trp mutations of CBM			
W249/251A	76.41 ± 41.88	35.53 ± 14.37	0.47 ± 0.34
W249/270A	66.60 ± 40.39	56.65 ± 24.52	0.85 ± 0.61
W251/270A	74.30 ± 43.25	76.63 ± 32.68	1.03 ± 0.76
Triple Trp mutation of CBM			
W249/251/270A	70.90 ± 31.50	28.67 ± 9.23	0.40 ± 0.29
W43A	16.71 ± 8.70	86.48 ± 21.20	5.18 ± 2.44
W224A	31.27 ± 12.07	68.62 ± 5.26	2.19 ± 1.26
W188A	17.72 ± 8.61	167.35 ± 39.00	9.44 ± 4.53
W192A	13.43 ± 6.23	219.35 ± 44.62	16.33 ± 7.17
E216A	11.08 ± 4.90	218.77 ± 39.97	19.74 ± 8.15

**Table 4:** Data for thermal shift assay comparison among i*Mt*Glu5, iΔCBM and mutants of i*Mt*Glu5 with or without CMC substrate

	Enzyme only (°C)	Bound to CMC (°C)	Δ*T*_i_
i*Mt*Glu5	87.1	91.1	4.0
iΔCBM	68.4	68.1	0.3
iW43A	83.9	85.2	1.3
iW224A	77.1	78.4	1.3
iW188A	87.6	89.1	1.5
iW192A	82.3	87.4	5.1
iE216A	84.5	90.3	5.8
iW249A	85.0	85.5	0.5
iW251A	78.4	78.7	0.3
iW270A	84.9	86.2	1.3
iW249/251A	74.8	74.8	0.0
iW249/270A	81.4	81.5	0.1
iW251/270A	75.8	75.7	0.1
iW249/251/270A	73.7	73.6	0.1
