# Supplementary material for: Synergic action of an inserted carbohydrate-binding module in a glycoside hydrolase family 5 endoglucanase
Source: Acta Crystallogr D Struct Biol. 2022 Apr 20;78(Pt 5):633–46. doi: 10.1107/S2059798322002601 (PMC9063844; doi:10.1107/S2059798322002601)
Supplement: Supplementary file 1 [file d-78-00633-sup1.pdf]

STRUCTURAL
BIOLOGY

Volume 78 (2022)

Supporting information for article:

Synergic action of an inserted carbohydrate-binding module in a glycoside hydrolase family 5 endoglucanase

Ting-Juan Ye, Kai-Fa Huang, Tzu-Ping Ko and Shih-Hsiung Wu

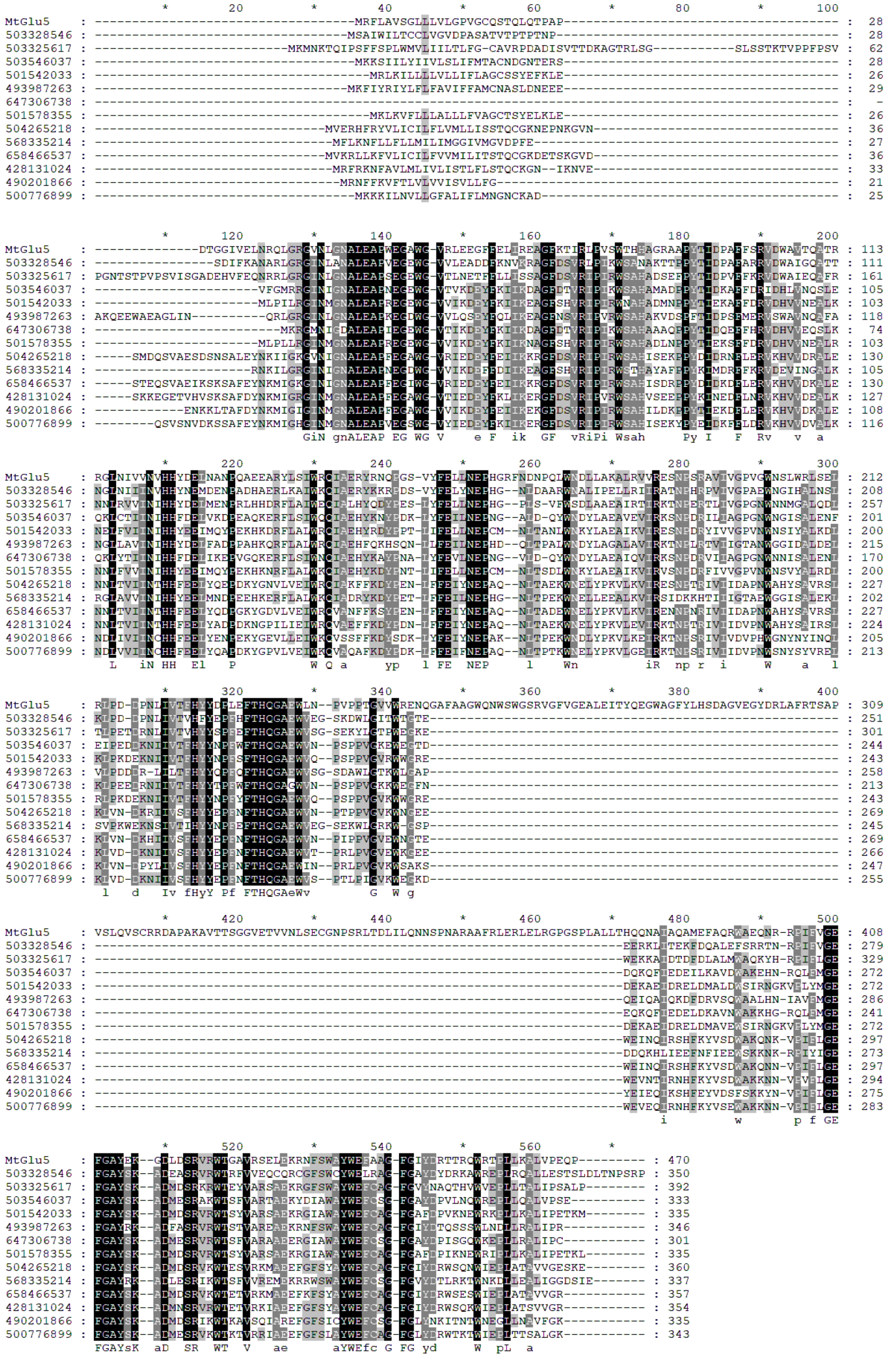

MtGlu5: *Miothermus taiwanensis* WR220

503328546: *Isosphaera pallida*

503325617: *Anaerolinea thermophila*

503546037: *Mahella australiensis*

501542033: *Dictyoglomus thermophilum*

493987263: *Caldithrix abyssi*

647306738: *Dictyoglomus turgidum*

501578355: *Caldicoprobacter oshimai*

504265218: *Fervidobacterium pennivorans*

568335214: *Thermotoga maritima*

658466537: *Thermotogae bacterium JGI 0000106-O11*

428131024: *Fervidobacterium gondwanense*

490201866: *Thermosipho africanus*

500776899: *Fervidobacterium nodosum*

Figure S1 Multiple-sequence alignment of *Mt*Glu5 compared with other GH5 cellulases.

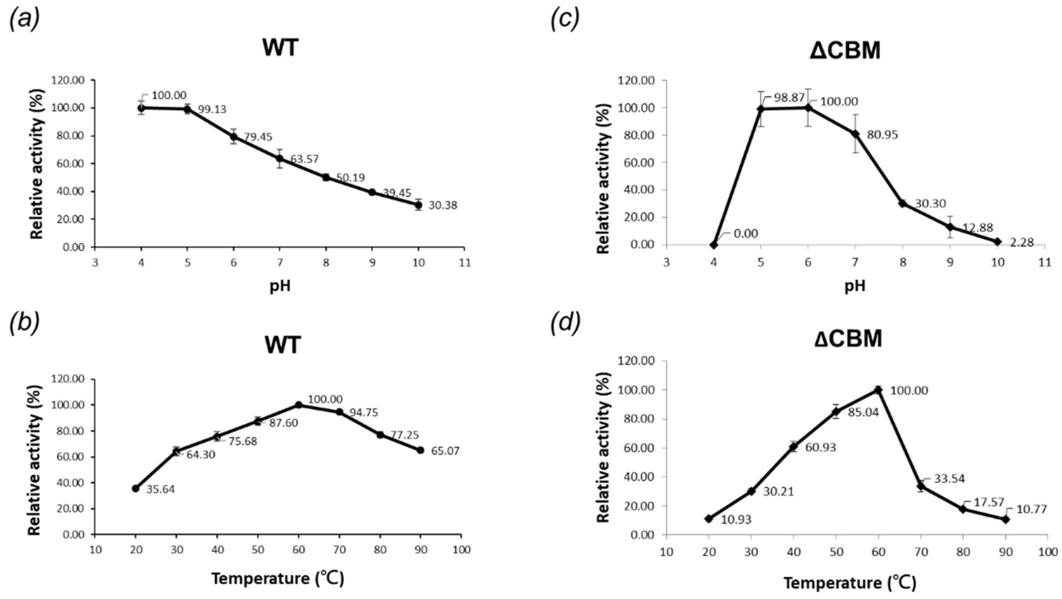

Figure S2 The effect of reaction pH and temperature on enzyme activity on CMC. Data are shown as the means ± SD of more than three replicates. Both WT *(a, b)* and ΔCBM *(c, d)* present an optimum condition at 60 °C and pH 5.

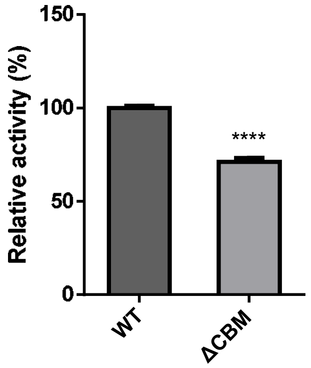

Figure S3 The activity comparison between *Mt*Glu5 (WT) and ΔCBM toward CMC. The specific activities of *Mt*Glu5 and ΔCBM toward CMC are 385 ± 5 IU and 275 ± 8 IU. Data are exhibited as the means ± SD of more than three replicates. **** indicates statistically significant at the level of $P < 0.0001$ compared to WT.

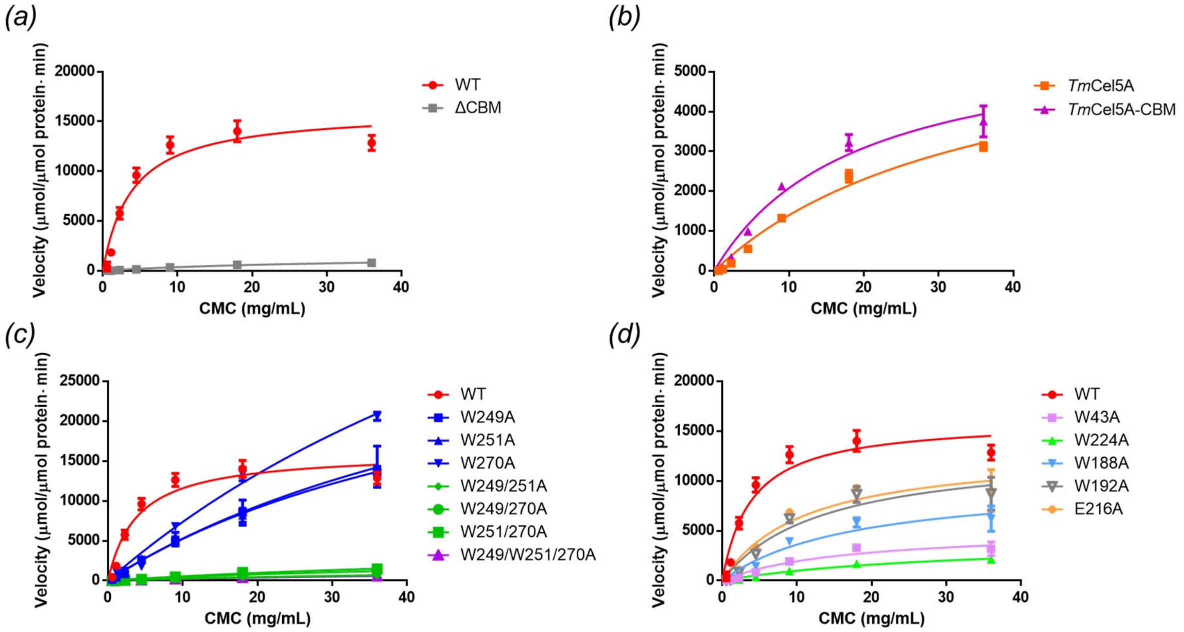

Figure S4 Reaction kinetics curves toward CMC. Comparison of the reaction kinetics curves among *Mt*Glu5 (WT) with ΔCBM *(a)*, *Tm*Cel5A with *Tm*Cel5A-CBM *(b)*, WT with Trp mutagenesis in CBM *(c)*, and WT with Trp mutagenesis in the catalytic domain *(d)*, respectively.

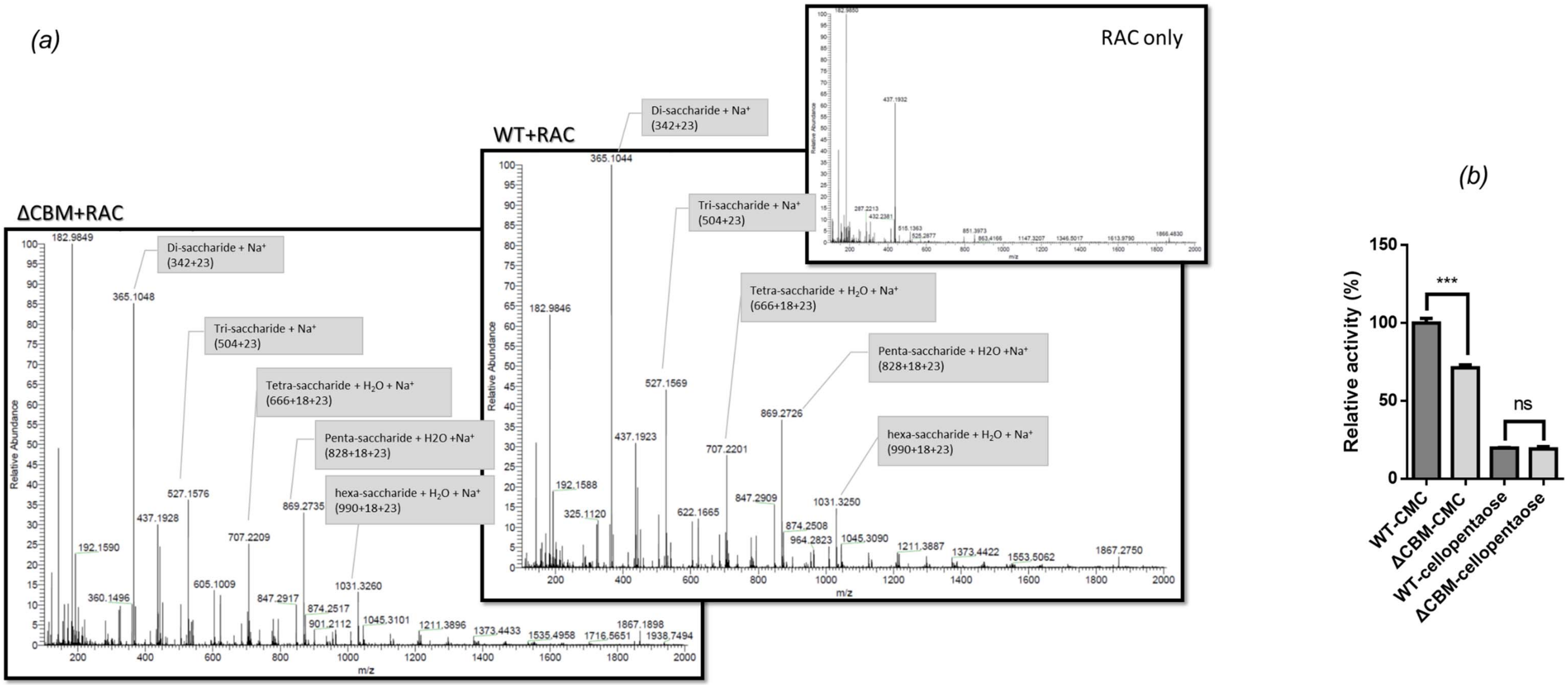

Figure S5 The catalytic centre integrity of WT and ΔCBM. *(a)* Hydrolyzed products are analyzed by mass spectrometry. Both WT and ΔCBM produce the same products after hydrolysis. m/z 182.98 and m/z 437.19 are considered contaminants in the background. Two enzymes were mixed with RAC and reacted for 24 hrs. The reactions were stopped by boiling. Then the pellet was removed, and hydrolyzed products remained in the supernatant fraction. Desalting was necessary before MS analysis. *(b)* Enzyme activities toward CMC and cellopentaose. Catalysis that would not use the CBM domain is demonstrated by using

cellopentaose as a substrate. The data show that the enzyme activity of *Mt*Glu5 (WT) and ΔCBM toward cellopentaose are almost the same, although lower than the activity toward CMC, indicating CDs are not perturbed by the CBM truncation. (Left-half panel) Enzyme activity toward CMC, *** indicates statistically significant at the level of $P < 0.001$ compared to *Mt*Glu5. (Right-half panel) Enzyme activity toward cellopentaose, ns represents no statistical significance as compared to *Mt*Glu

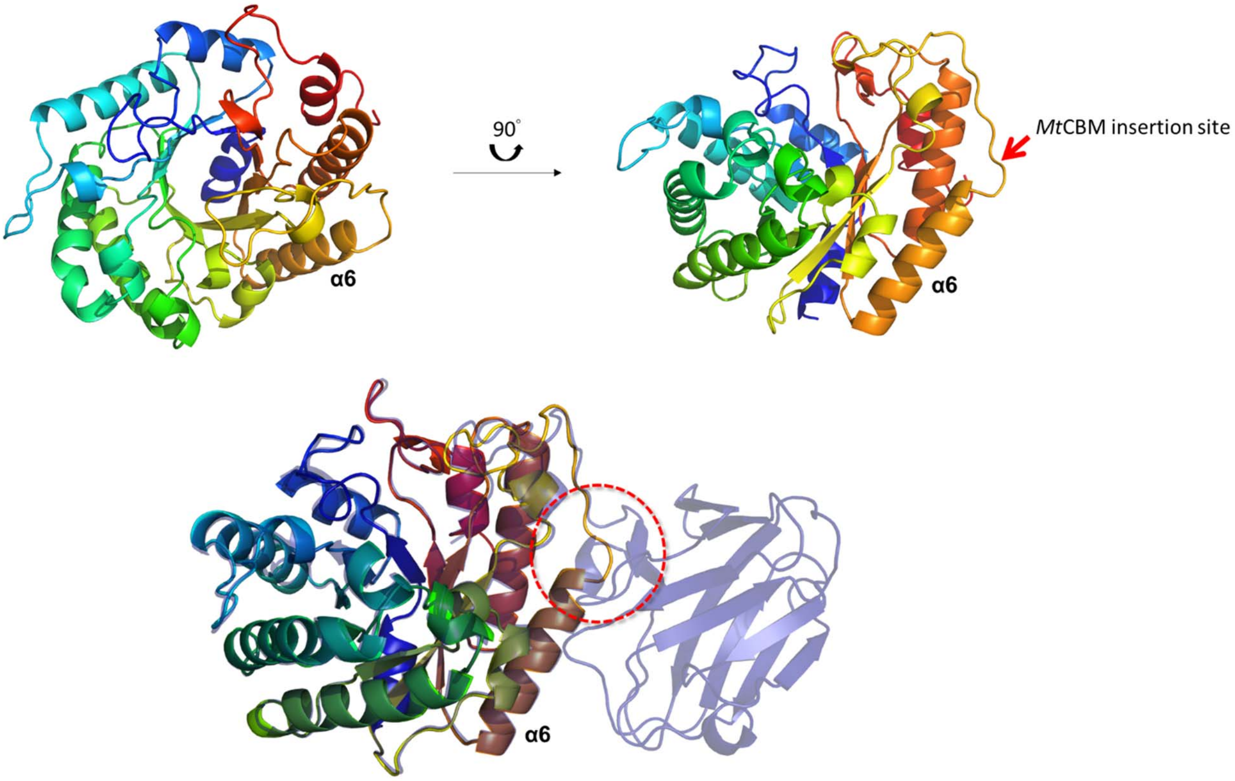

Figure S6 The structure of ΔCBM ramped from the N-terminus (blue) to the C-terminus (red) (PDB: 7VT5). The apo-form ΔCBM structure displayed an intact TIM-barrel fold. The red arrow indicates the location of *Mt*CBM insertion site (a.a 235 of *Mt*Glu5). Superimposition of *Mt*Glu5 and ΔCBM highlights the slight change in helix α6.

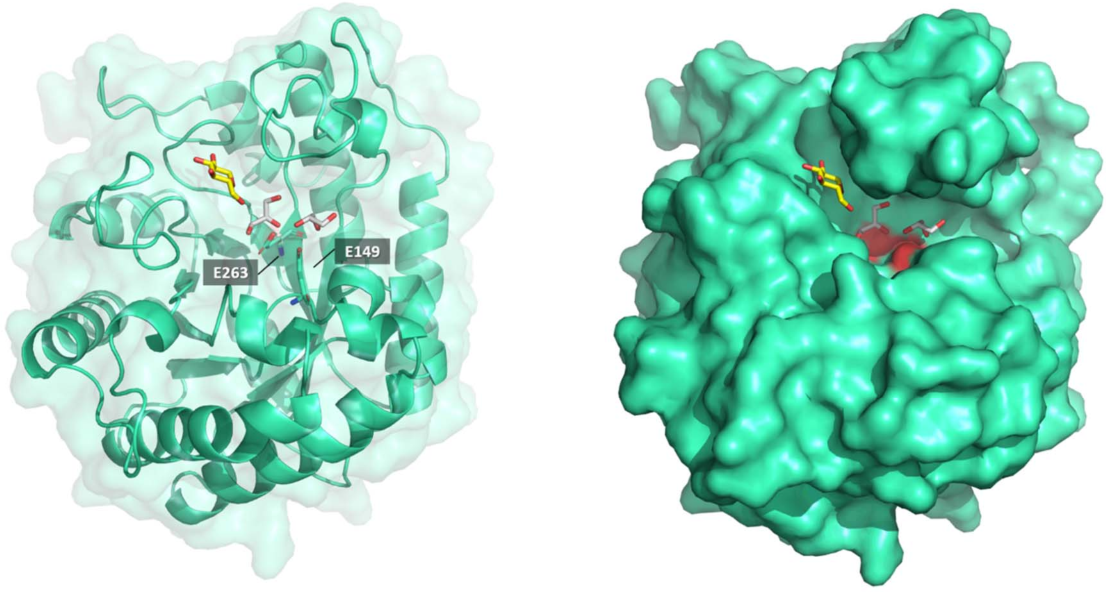

Figure S7 The structure of ΔCBM in complex with glucose (PDB: 7VT6). Glucose is shown in a stick form (yellow) whereas two glycerol molecules that occupied the catalytic site are shown in white sticks.

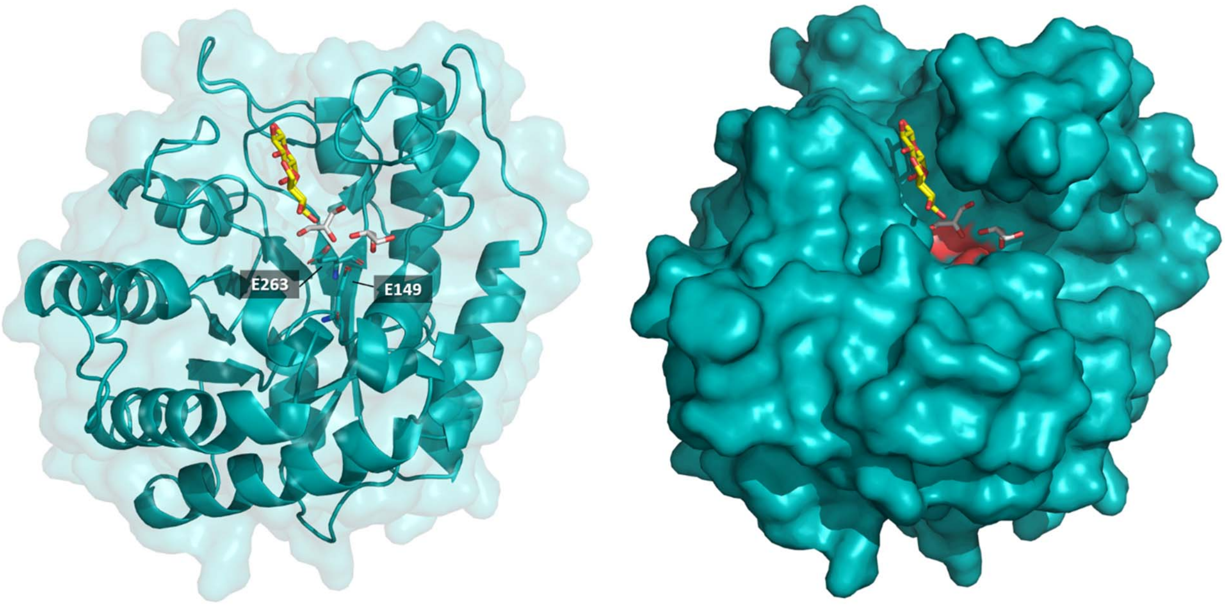

Figure S8 The structure of ΔCBM in complex with cellobiose (PDB: 7VT7). Cellobiose is shown in a stick form (yellow) whereas two glycerol molecules that occupied the catalytic site are shown in white sticks.

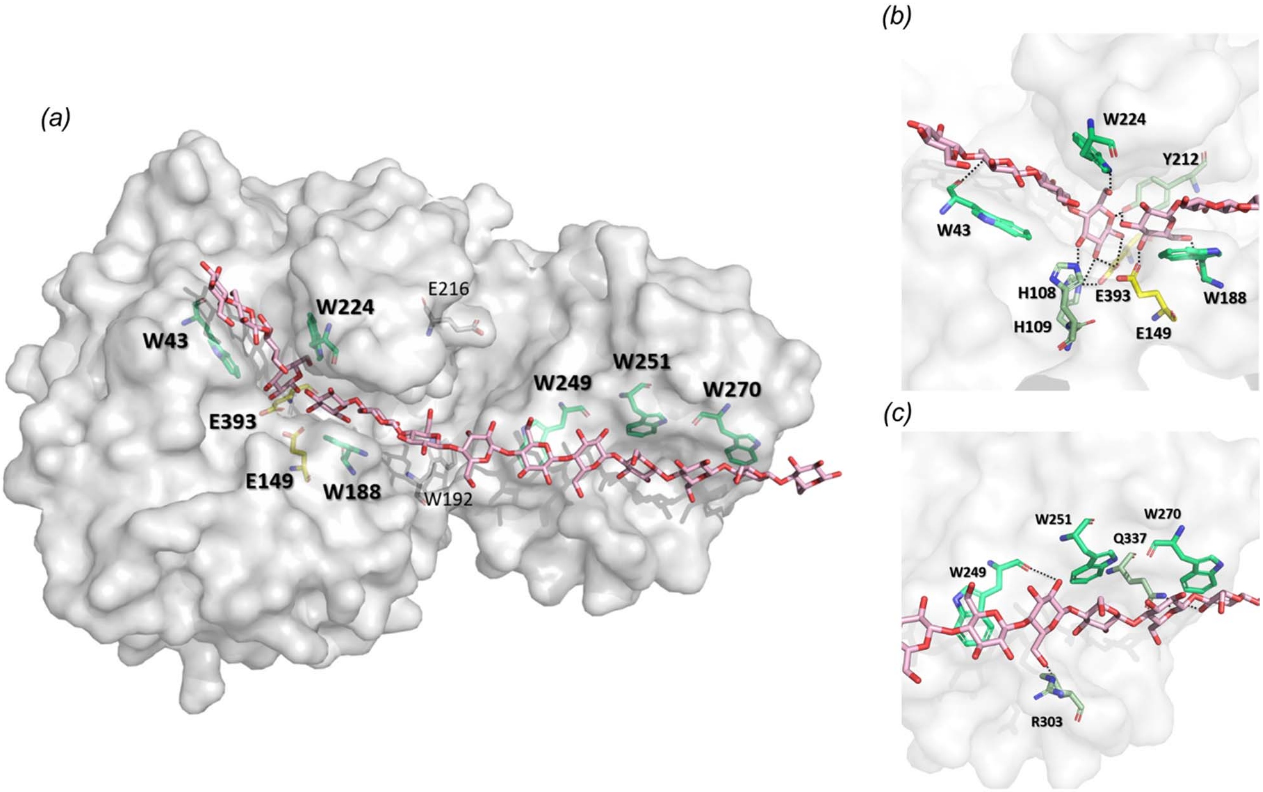

Figure S9 Substrate binding model of *Mt*Glu5 depicted in surface structure. *(a)* A 14-sugar-long single polysaccharide chain, shown in stick format (pink), is bound along the putative binding groove full of Trp residues (lime green) on the cleft surface. In this model, the polysaccharide chain is stabilized by the arrangement of the aromatic residues in the cleft of *Mt*Glu5. The catalytic residues, E149 and E393, are shown in yellow and stick representation while the less essential residues, W192 and E216, are shown in white. The binding model is superimposed onto *Tm*Cel5A in complex with cellotetraose (PDB: 3azt) and CBM29-2 in complex with cellohexaose (PDB: 1W8T) then 14-polysaccharides are put in by *coot*. Energy minimization is simulated by the software YASARA Energy Minimization server. Snapshots of hydrogen bonds (black dashed lines) at catalytic pocket *(b)* and CBM binding surface *(c)*. The surface Trp residues are shown as described and the other active-site residues are shown in olive green. *(b)* In the catalytic pocket, W43, W224, and W188 show not only steaking interaction but a direct hydrogen bond to the substrate. *(c)* W249, R303, and Q337 residues make direct interactions with the substrate in the same way as CBM29-2.

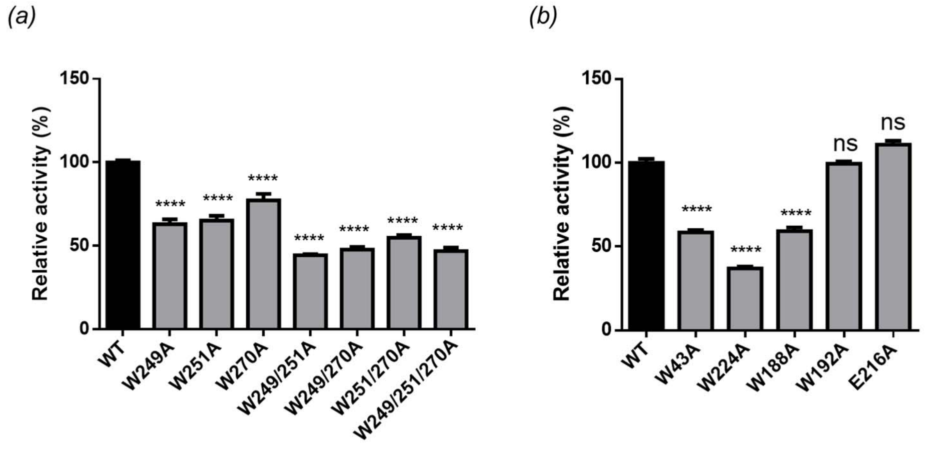

Figure S10 The activity comparison between *Mt*Glu5 (WT) and Trp mutants on CMC. Data are exhibited as the means ± SD of more than three replicates. **** indicates statistical significance at the level of $P < 0.0001$ compared to WT, and *ns* is no significant differences compared to WT, respectively. *(a)* Activity comparison among WT and CBM mutants. *(b)* Activity comparison among WT and CD mutants.

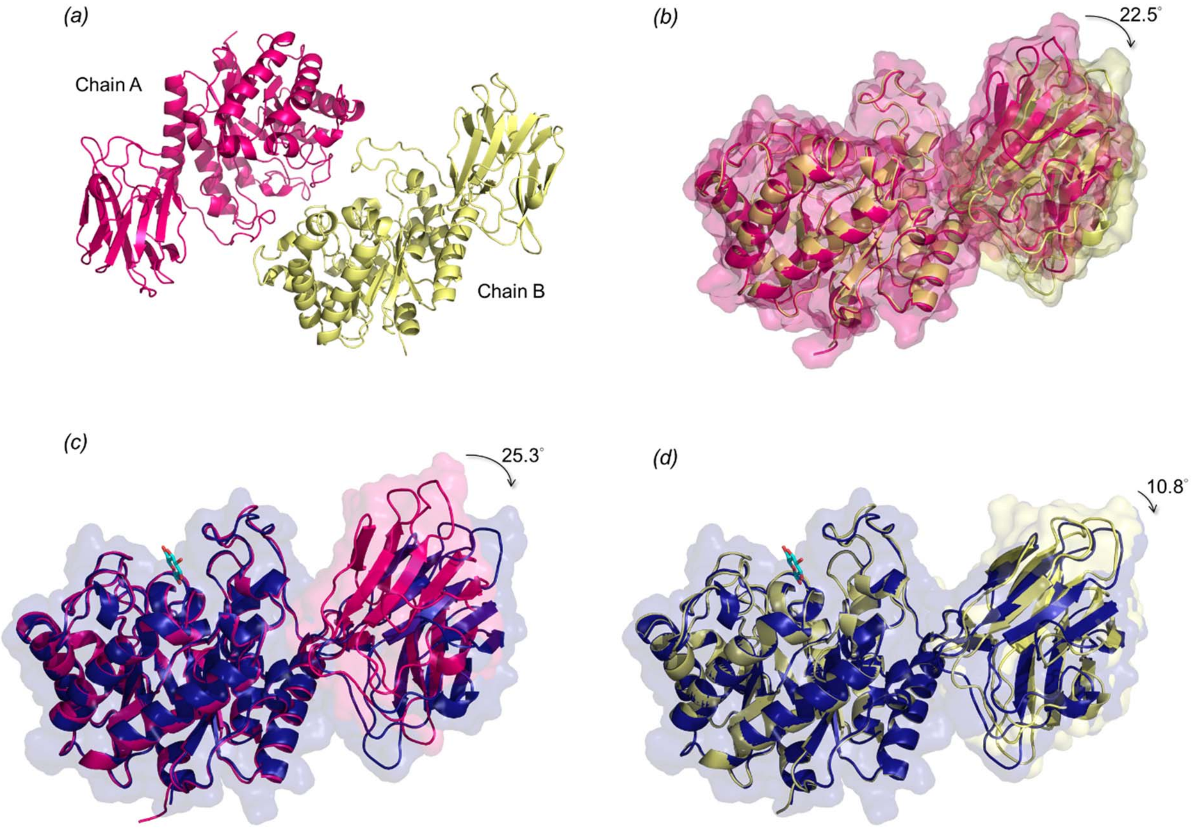

Figure S11 Structure comparison of i*Mt*Glu5 (PDB: 7VT4) with *Mt*Glu5 (PDB: 7VT8). *(a)* crystal structure of i*Mt*Glu5 shows dimer in the asymmetric unit. *(b)* By overlaying two chains of i*Mt*Glu5, the CBMs of the two i*Mt*Glu5 molecules differ by a rigid-body rotation of 22.5°. *(c)* Superimposition of glucose-bound *Mt*Glu5 and i*Mt*Glu5 chain A and chain B *(d)*.

Table S1 List of primers.

Construct	Primers (5’ to 3’)
*Mt*Glu5	F1: TATGATGGGCTGCCAATCCACC<br>F2: TGATGGGCTGCCAATCCACC<br>R1: AGCTTCGGTTGCTCCGGTACAA<br>R2: TCGGTTGCTCCGGTACAA
i*Mt*Glu5<br>ΔCBM	F1: TCGTGGGGCAGTTCGGAGCCTAC<br>F2: CCTACGAGAAGGGCGATCT<br>R1: CTCCGAACTGCCCCACGAAGA<br>R2: AGATGGGGCGCCGGTTCT
W249A<br>iW249A	F1: CAGAACGCATCCTGGGGCAGCCGG<br>F2: AGCCGGGTCGGTTTTGTGGG<br>R1: GCCCCAGGATGCGTTCTGCCAGCCCG<br>R2: CCAGCCCGCCGCGAAGGC
W251A<br>iW251A	F1: TGGTCCGCAGGCAGCCGGGTCG<br>F2: GTCGGTTTTGTGGGTGAAGCT<br>R1: CCGGCTGCCTGCGGACCAGT<br>R2: GTTCTGCCAGCCCGCCGC
W270A<br>iW270A	F1: AAGGCGCAGCCGGGTTCTATTTG<br>F2: ATTTGCACTCGGACGCTGGG<br>R1: AGAACCCGGCTGCGCCTTCCTGGTA<br>R2: CCTGGTAGGTGATCTCTAAAGCT
W249/251A<br>iW249/251A	F1: TCCGCAGGCAGCCGGGTCGGTTTTGT<br>F2: GTCGGTTTTGTGGGTGAAGCTT<br>R1: CCGGCTGCCTGCGGATGCGTTCTGCCAGC<br>R2: TGCGTTCTGCCAGCCCGCCGCGAA
W249/270A<br>iW249/270A	Same as W249A using W270 as a template
W251/270A	Same as W251A using W270 as a template

iW251/270A	
W249/251/270A<br>iW249/251/270A	Same as W249/251A using W270 as a template
W43A<br>iW43A	F1: GGGGCCGCAGGGGTCAGGCT<br>F2: CTGGAGGAAGGGTTTTTCGAGCTG<br>R1: CCTGACCCCTGCGGCCCCTTCCC<br>R2: TTCCCAGGGGGCCTCGAGGGC
W188A<br>iW188A	F1: GTGGGTGCCAACTCGCTGTGGCGGCTG<br>F2: TGGCGGCTGTCCGAGCTGCG<br>R1: CAGCGAGTTGGCACCCACCGGCCCC<br>R2: CGGCCCCACAATCACCGCCC
W192A<br>iW192A	F1: TCGCTGGCCCGGCTGTCCGAGCTG<br>F2: GAGCTGCGGCTGCCGGACGAT<br>R1: GGACAGCCGGGCCAGCGAGTTCCAAC<br>R2: GTTCCAACCCACCGGCCCCAC
E216A<br>iE216A	F1: CCCCTGGCGTTCACCCACCAGG<br>F2: CAGGGGGCTGAGTGGCTC<br>R1: GTGGGTGAACGCCAGGGGATCGTA<br>R2: ATCGTAGTAGTGGAAGGTAACGA
W224A<br>iW224A	F1: GCTGAGGCTCTCAACCCCGTTCCACC<br>F2: GTTCCACCTACCGGTGTGGTCTGG<br>R1: GGGGTTGAGAGCCTCAGCCCCCTG<br>R2: CCCCTGGTGGGTGAACTCCAGGGG
ΔCBM	F1: CACCAGCAAAACGCCATAGC<br>F2: GCCCAGGCCATGGAGTTT<br>R1: TATGGCGTTTTGCTGGTGCCAGACCACACCGGTAGG<br>R2: CCAGACCACACCGGTAGG
g*Mt*CBM	F1: TATGTCCCCTATACTAGGTTATTGG<br>F2: TGTCCCCTATACTAGGTTATTGG<br>R1: AGCTTGGTCAGCAAAGCCAGCGG

	R2: TGGTCAGCAAAGCCAGCGG<br>GST-F: TGTCCCCTATACTAGGTTATTGG<br>GST-R: CACCCTGGTTCTCACGGGATCCACGCGGAACCAG<br>CBM-F: CGTGAGAACCAGGGTGCGTTC<br>CBM-R: TGGTCAGCAAAGCCAGCGG
GST	F1: TATGATGTCCCCTATACTAGGTT<br>F2: TGATGTCCCCTATACTAGGTT<br>R1: AGCTTTTTTGGAGGATGGTCGCC<br>R2: TTTTTGGAGGATGGTCGCC
*Tm*Cel5A	F1: TATGATGGGTGTGGATCCGTTC<br>F2: TGATGGGTGTGGATCCGTTC<br>R1: AGCTTTTCGATGCTGTCGCCACC<br>R2: TTTCGATGCTGTCGCCACC
*Tm*Cel5A-CBM	F1: TATGATGGGTGTGGATCCGTTC<br>F2: TGATGGGTGTGGATCCGTTC<br>R1: AGCTTTTCGATGCTGTCGCCACC<br>R2: TTTCGATGCTGTCGCCACC<br>Cel5A-F1: TGATGGGTGTGGATCCGTTC<br>Cel5A-R1: GCCCCATTTACGACCCAGCCACTTCTC<br>CBM-F: CTGGGTCGTAAATGGGGCCGTGAGAACCAGGGTGCGTT<br>CBM-R: CTTTTGATCGTCCGGGCTGGTCAGCAAAGCCAGCGG<br>Cel5A-F2: AGCCCGGACGATCAAAAGCACCTGA<br>Cel5A-R2: TTTCGATGCTGTCGCCACC
i*Tm*Cel5A<br>i*Tm*Cel5A-CBM	F1: ATCGGCCAATTCGGCGCGTATC<br>F2: TATCGTAAGGCGGATCTGGAAAGCC<br>R1: CGCGCCGAATTGGCCGATGTAAATC<br>R2: GTAAATCGGACGTTTGTTTTTCTTGC

Table S2 The processivity of *Mt*Glu5 towards RAC.

	Enzyme	Reducing sugar (%)		Ratio
		Supernatant	Insoluble	Sol./Insol.
RAC	*Mt*Glu5	72.6	27.4	2.65
	ΔCBM	62.5	37.5	1.67

The processivity is presented as the ratio of the reducing ends in the soluble part to the one in the insoluble part after hydrolysis. We tested the classic methods to verify the processivity by determining the ratio of soluble reducing ends to insoluble reducing sugar ends products using both RAC and filter paper (Data not shown) as substrates, both *Mt*Glu5 and ΔCBM present the ratio of around 2.65 and 1.67, by the criterion exhibited in the paper (Irwin *et al.*, 1993). Therefore *Mt*Glu5 is clearly an endo-glucanase.
